# Restoration of Olfactory Memory in *Drosophila* Overexpressing Human Alzheimer’s Disease Associated Tau by Manipulation of L-Type Ca^2+^ Channels

**DOI:** 10.3389/fncel.2019.00409

**Published:** 2019-09-10

**Authors:** James P. Higham, Sergio Hidalgo, Edgar Buhl, James J. L. Hodge

**Affiliations:** School of Physiology, Pharmacology and Neuroscience, University of Bristol, Bristol, United Kingdom

**Keywords:** tau, tauopathy, Alzheimer’s disease, memory, L-type Ca2+ channels, *Drosophila*, GCaMP Ca2+ imaging

## Abstract

The cellular underpinnings of memory deficits in Alzheimer’s disease (AD) are poorly understood. We utilized the tractable neural circuits sub-serving memory in *Drosophila* to investigate the role of impaired Ca^2+^ handling in memory deficits caused by expression of human 0N4R isoform of tau which is associated with AD. Expression of tau in mushroom body neuropils, or a subset of mushroom body output neurons, led to impaired memory. By using the Ca^2+^ reporter GCaMP6f, we observed changes in Ca^2+^ signaling when tau was expressed in these neurons, an effect that could be blocked by the L-type Ca^2+^ channel antagonist nimodipine or reversed by *RNAi* knock-down of the L-type channel gene. The L-type Ca^2+^ channel itself is required for memory formation, however, *RNAi* knock-down of the L-type Ca^2+^ channel in neurons overexpressing human tau resulted in flies whose memory is restored to levels equivalent to wild-type. Expression data suggest that *Drosophila* L-type Ca^2+^ channel mRNA levels are increased upon tau expression in neurons, thus contributing to the effects observed on memory and intracellular Ca^2+^ homeostasis. Together, our Ca^2+^ imaging and memory experiments suggest that expression of the 0N4R isoform of human tau increases the number of L-type Ca^2+^ channels in the membrane resulting in changes in neuronal excitability that can be ameliorated by *RNAi* knockdown or pharmacological blockade of L-type Ca^2+^ channels. This highlights a role for L-type Ca^2+^ channels in tauopathy and their potential as a therapeutic target for AD.

## Introduction

The accumulation of the microtubule-associated protein tau (MAPT) within central neurons is a key histopathological feature of Alzheimer’s disease (AD) (Kaufman et al., 2018; Nisbet and Gotz, 2018). Post-mortem AD brain samples contain the hallmark accumulation of extracellular amyloid beta (Aβ) plaques and intracellular neurofibrillary tangles (NFTs) of hyperphosphorylated tau, resulting in neurodegeneration and brain atrophy. The accumulation of tau is more correlated with the progression of AD pathology and symptoms than the magnitude of Aβ plaques (Arendt et al., 2016). However, the mechanisms by which tau disrupts neuronal function, and consequently behavior, are not clear. Tau exists in six major isoforms formed by alternative splicing of exons 2 and/or 3 (0N, 1N or 2N) and 10 (3R or 4R) of the MAPT-17 gene (Buee et al., 2000). There is some evidence that 4R isoforms are upregulated in post-mortem brains, particularly within the hippocampus of AD patients (Boutajangout et al., 2004; Espinoza et al., 2008; Hasegawa et al., 2014), and show stronger affinity binding to tubulin and aggregation than 3R forms (Arendt et al., 2016). However, both 3R and 4R isoforms are present in NFTs, suggesting both play a role in pathology. The expression of 0N4R tau in model organisms can yield neuronal and behavioral dysfunction prior to the onset of neurodegeneration (Wittmann et al., 2001; Mershin et al., 2004). This suggests that tau’s detrimental effect on neurons is sufficient to drive behavioral changes before neuronal death.

Patients with AD display memory impairment, and once the onset of neurodegeneration has occurred, treating the symptoms of AD becomes exceedingly difficult, so targeting the earlier signs of neuronal dysfunction is likely to be more efficacious. To this end, an understanding of how tau influences neuronal function is required. Disrupted calcium (Ca^2+^) homeostasis including increased Ca^2+^ levels causing excitotoxicity has been posited as a key pathophysiology in AD, which may underpin the early stages of disease and precipitate neurodegeneration (Mattson and Chan, 2001; Brzyska and Elbaum, 2003; Canzoniero and Snider, 2005). Recent work suggests that these changes in neuronal excitability and Ca^2+^ signaling provide an important link between Aβ and tau pathology and disease progression (Spires-Jones and Hyman, 2014; Wu et al., 2016), but exactly how remains unknown. Different rodent models of tauopathy recapitulate some features of AD pathology including changes in excitability (Booth et al., 2016a, b), increased Ca^2+^ signaling (Wang and Mattson, 2014), neurodegeneration (Spillantini and Goedert, 2013), and impaired synaptic plasticity and memory (Arendt et al., 2016; Biundo et al., 2018). It is not clear what the source of increased Ca^2+^ is, however, increased levels of Ca^2+^ channels, such as the L-type voltage-gated Ca^2+^ channel (Ca_V_1), have been shown to be upregulated in rodent models of AD, with blockers, such as nifedipine, being effective in trails to prevent the cognitive decline that occurs in AD (Coon et al., 1999; Anekonda et al., 2011; Goodison et al., 2012; Nimmrich and Eckert, 2013; Daschil et al., 2015). The role of voltage-gated Ca^2+^ channels in memory or AD has not been studied in *Drosophila*. *Drosophila* contains three different voltage-gated Ca^2+^ channel genes including the *DmCa1D* or *Ca-α1D* gene that encodes a high voltage-activated current and is equivalent to the vertebrate Ca_V_1.1-1.4 genes that encode L-type Ca^2+^ channels (Worrell and Levine, 2008).

Alzheimer’s disease and tauopathies have been modeled in *Drosophila* (Iijima-Ando and Iijima, 2010; Wentzell and Kretzschmar, 2010; Papanikolopoulou and Skoulakis, 2011), with targeted neuronal expression of human 0N4R tau causing neurodegeneration, shortened lifespan, circadian, sleep and motor deficits (Wittmann et al., 2001; Kerr et al., 2011; Sealey et al., 2017; Buhl et al., 2019; Higham et al., 2019). Furthermore, during development at the larval neuromuscular junction, motor neurons overexpressing human 0N3R or 0N4R caused a reduction in size and irregular and abnormally shaped synaptic terminals, a reduction in endocytosis and exocytosis and a reduction in high frequency synaptic transmission (Chee et al., 2005; Zhou et al., 2017). Also, while expression of tau 0N3R in the adult giant fiber system caused increased failure rate at high frequency stimulation, expression of 0N4R caused defects in stimulus conduction, response speed and conduction velocity (Kadas et al., 2019).

Learning and memory deficits can also be assessed in *Drosophila* using aversive olfactory classical conditioning (Malik et al., 2013; Malik and Hodge, 2014). Different mushroom body (MB) neuropils underpin specific phases of memory and are redundant during others (Pascual and Preat, 2001; Krashes et al., 2007; Davis, 2011). MB neurons send axons that terminate in lobed structures, with memory acquisition being mediated by the α′β′ lobe neurons and memory-storage by the αβγ neurons. In addition, a number of additional pairs of neurons innervate or are innervated by the MB and mediate different aspects of memory. For instance, the amnesiac expressing dorsal paired medial (DPM) and anterior paired lateral (APL) neurons are thought to consolidate and stabilize labile memory (Pitman et al., 2011). Finally, the MB innervate a pair of M4/6 MB output neurons via a cholinergic synapse, with optogenetic manipulation of the M4/6 neurons switching between appetitive and aversive memory (Barnstedt et al., 2016). Previous work has demonstrated that expression of tau in γ neurons caused a reduction in learning and 1.5 h memory in 3–5 days old young flies which were shown to still have their γ neurons intact, prior to their degeneration around day 45 (Mershin et al., 2004). Moreover, pan-neuronal 0N4R expression caused learning and long-term memory loss while 0N3R tau expression failed to do so (Sealey et al., 2017).

Therefore, *Drosophila* expressing human tau can be used to study AD relevant behaviors and their underlying neuronal mechanism, with loss of memory being possible prior to neurodegeneration. Although tau-mediated changes in neuronal properties are likely underpinning the memory impairment observed in *Drosophila* AD models, these changes have not been extensively studied in *Drosophila* MB neurons.

In this study, we determined the effect of human 0N4R tau expression in different neuropils of the *Drosophila* memory circuit on one-hour memory. We measured the effect of 0N4R tau on MB output neuron mediated memory and Ca^2+^ signaling and described a potential interaction between 0N4R tau with L-type voltage-gated Ca^2+^ channels which may underpin memory impairment.

## Results

**FIGURE 1:**
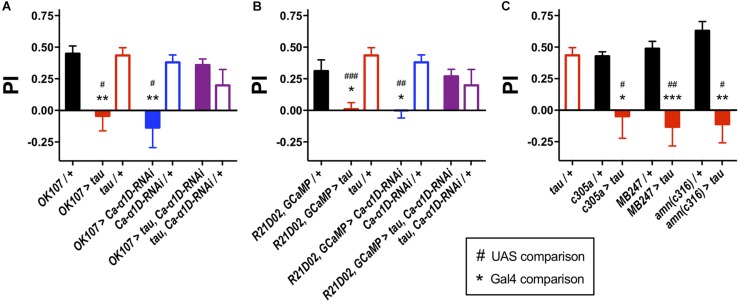
Knock-down of L-type Ca^2+^ channels rescued tau-induced memory defects. **(A)** Tau expression in the MB (*OK107-Gal4*) ablated one-hour memory (*n* = 7 experiments with 50–100 flies each, red bar), as did the expression of an *RNAi* to the gene encoding the L-type Ca^2+^ channel alpha subunit (*Ca-*α*1D-RNAi*, *n* = 8, blue bar) compared to both Gal4 (*n* = 12, black bar) and UAS controls (tau, *n* = 11, open red bars; *Ca-*α*1D-RNAi*, *n* = 9, open blue bars). Co-expression of both transgenes (*n* = 12, purple bar) resulted in flies with a memory performance equivalent to that of control animals, as did the UAS controls (*n* = 4, open purple bar). **(B)** Likewise, expression of tau (*n* = 9) or *Ca-*α*1D-RNAi* (*n* = 6) in the MB efferent M4/6 neurons (*R21D02-Gal4*, *n* = 10) abolished memory. Memory was restored to wild-type levels by co-expression of both transgenes (*n* = 15). **(C)** Expression of tau in neuronal subpopulations of the MB impaired memory. Expression was driven in the α′β′ neurons (*c305a-Gal4*, *n* = 6 control, *n* = 4 tau), αβγ neurons (*MB247-Gal4*, *n* = 10 control, *n* = 7 tau) and dorsal paired medial neurons (*amn(c316)-Gal4*, *n* = 5 control, *n* = 4 tau). Data analyzed with Kruskal–Wallis test with Dunn’s *post hoc* tests **(A,C)** or with one-way ANOVA with Dunnett’s *post hoc* tests **(B)**. ^*/#^*p* < 0.05, ^**/##^*p* < 0.01, and ^***/###^*p* < 0.001. Note that *UAS/* + controls are the same for each panel.

To determine the effect of human tau 0N4R on memory, we targeted expression of the transgene to different neuronal populations of the *Drosophila* memory circuit and performed one-hour olfactory aversive conditioning. Driving expression of a human tau 0N4R transgene (Wittmann et al., 2001; Kerr et al., 2011; Higham et al., 2019) in the entire MB (*OK107-Gal4* (Malik et al., 2013), p_Gal__4_ = 0.004, p_UAS_ = 0.01, Figure 1A) yielded memory deficient flies. Similarly, when expressed in the memory-relevant M4/6 MB output neurons (Barnstedt et al., 2016), *R21D02-Gal4, GCaMP6f* > *tau* flies displayed greatly reduced memory performance compared to control counterparts (p_Gal__4_ = 0.02, p_UAS_ < 0.001, Figure 1B). We also found that tau caused a significant reduction in one-hour memory when expressed in the α′β′ MB neurons (*c305a-Gal4* (Krashes et al., 2007), p_Gal__4_ = 0.02, p_UAS_ = 0.01, Figure 1C), which mediate memory acquisition and in the memory-storing αβγ neurons (*MB247-Gal4* (Krashes et al., 2007), p_Gal__4_ < 0.001, p_UAS_ = 0.003) and dorsal paired medial (DPM) neurons (*amn*(*c316)-Gal4*, p_Gal__4_ = 0.002, p_UAS_ = 0.03), which consolidate and stabilize labile memory (Pitman et al., 2011). All animals used for aversive conditioning were aged 2–5 days and displayed naïve avoidance of shock (Supplementary Table S1). Likewise, all genotypes showed similar naïve avoidance of the odors used for olfactory conditioning (Supplementary Table S1). Thus, all genotypes were able to detect both odors and shock, verifying that memory defects were *bone fide* and not attributable to a sensorimotor defect.

**FIGURE 2:**
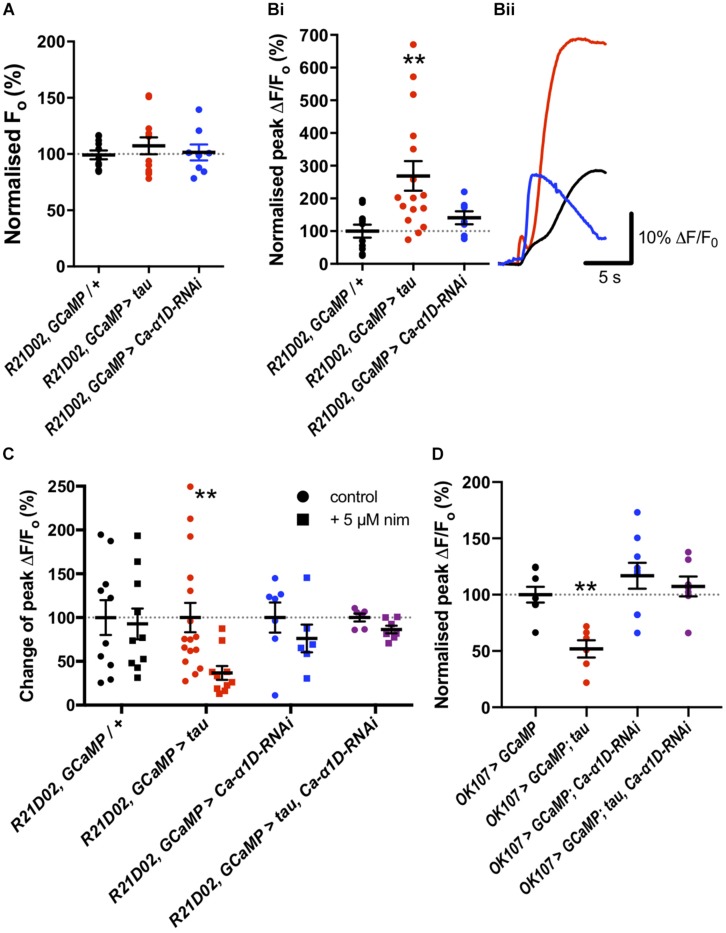
Tau expression elevated nimodipine-sensitive Ca^2+^ channels in mushroom body (MB) and M4/6 neurons. **(A)** Basal Ca^2+^ levels were no different between control neurons and those expressing tau or *Ca-*α*1D-RNAi*. **(Bi)** However, the peak magnitude of Ca^2+^ transients were greater in the tau-expressing neurons than in control or *Ca-*α*1D-RNAi*-expressing neurons. **(Bii)** GCaMP6f relative fluorescence changes over time traces of M4/6 neurons in response to 100 mM KCl for control (black trace), tau (red trace) and *Ca-*α*1D-RNAi* (blue trace). **(C)** Ca^2+^ transients in control and *Ca-*α*1D-RNAi*-expressing neurons were not sensitive to the L-type Ca^2+^ channel blocker nimodipine (black and blue symbols, respectively). Driving tau expression in M4/6 neurons conferred sensitivity of Ca^2+^ transients to nimodipine (red symbols). The effect of tau on Ca^2+^ transient nimodipine sensitivity was ablated by co-expression of tau and *Ca-*α*1D-RNAi* in these neurons (purple symbols). Control for each genotype normalized to 100%. **(D)** Tau expression in the MB reduced peak fluorescence following a high KCl challenge; an effect reversed by co-expression of *Ca-*α*1D-RNAi*. Data analyzed with one-way ANOVA with Dunnett’s *post hoc* tests panels **(A,D)**, with Kruskal–Wallis test with Dunn’s *post hoc* tests panel **(B)** or with two-way ANOVA with Sidak’s *post hoc* tests panel **(C)**. ^*/#^*p* < 0.05, ^**/##^*p* < 0.01, and ^***/###^*p* < 0.001. Note that data for *OK107* > *GCaMP* and *OK107* > *GCaMP, tau* are reproduced from Higham et al. (2019).

To assess changes in Ca^2+^ handling which may underpin behavioral dysfunction, we imaged the M4/6 neurons in whole *ex vivo* brains using GCaMP6f as these neurons are pertinent to memory and single neurons can be visualized (Barnstedt et al., 2016). There did not appear to be a difference in baseline fluorescence of these neurons between control and tau-expressing animals (*p* = 0.5, Figure 2A), suggesting no effect on basal Ca^2+^ handling, assuming comparable GCaMP6f expression between genotypes. Bath application of high potassium chloride (KCl) concentration resulted in a robust and transient elevation in fluorescence which could be ameliorated by removing Ca^2+^ from the external solution (12.8 ± 1.0% of control, *p* = 0.003) or by adding cadmium (200 μM, 11.75 ± 11.75% of control, *p* = 0.009), a general blocker of *Drosophila* voltage-gated Ca^2+^ channels (Ryglewski et al., 2012), to the external solution (Supplementary Figure S2A). This indicated that the transient relies upon Ca^2+^ influx through voltage-gated Ca^2+^ channels, or that cadmium blocked presynaptic Ca^2+^ channels and, consequently, neurotransmitter release and activation of M4/6 neurons.

KCl-evoked Ca^2+^ transients in tau-expressing M4/6 neurons were almost three-fold greater than in control neurons (268.9 ± 45.2% of control, *p* = 0.005, Figure 2B). Since previous reports have documented the potential involvement of L-type Ca^2+^ channels in AD (Wang and Mattson, 2014; Coon et al., 1999), we tested whether the change in magnitude of Ca^2+^ transients in M4/6 neurons was dependent on these channels by applying the clinically-used L-type channel-selective blocker nimodipine (Nimmrich and Eckert, 2013; Terada et al., 2016). The addition of 5 μM nimodipine to the external solution had no effect on the magnitude of Ca^2+^ transients in control brains (*p* > 0.9, Figure 2C). However, the peak of the transient in tau-expressing neurons was sensitive to nimodipine and was reduced to a magnitude indistinguishable from control (98.9 ± 21.1% of control, *p* > 0.9). The elevated Ca^2+^ influx seen in these neurons may, therefore, be due to augmented L-type Ca^2+^ channels. To further investigate which type of voltage-gated Ca^2+^ channel mediated the Ca^2+^ influx, we also tested the effect of addition of the L-type and T-type Ca^2+^ channel blocker, amiloride (1 mM), to nimodipine on the KCl-induced Ca^2+^ transient, as this compound greatly reduced Ca^2+^ currents in cultured embryonic *Drosophila* giant neurons (Peng and Wu, 2007). Amiloride did not significantly reduce the peak of the Ca^2+^ transient in M4/6 neurons (*p* = 0.2, Supplementary Figure S2B). The lack of effect of amiloride likely indicates developmental or cell-specific differences in Ca^2+^ channel expression or that the voltage-gated Ca^2+^ channels blocked by amiloride largely overlap with nimodipine which do not contribute significantly to the Ca^2+^ transient in wild-type M4/6 neurons.

To corroborate our pharmacological data, we tested the nimodipine sensitivity of Ca^2+^ transients in M4/6 neurons co-expressing tau and an *RNAi* to the L-type Ca^2+^ channel gene (*UAS-Ca-α1D-RNAi*), that has been shown to reduce L-type Ca^2+^ channel currents and protein levels by over 75% in *Drosophila* neurons (Kadas et al., 2017). Ca^2+^ transients in these neurons displayed a much reduced, and statistically insignificant (*p* > 0.05, Figure 2C), block by nimodipine compared to transients in neurons expressing tau alone (13.2% vs. 62.3% reduction in peak). This data shows that the elevated Ca^2+^ transients in tau-expressing M4/6 neurons rely on L-type Ca^2+^ channels. As L-type channels negatively regulate neuronal excitability (Worrell and Levine, 2008), *RNAi* mediated reduction of L-type Ca^2+^ channels increases neuronal activity (Kadas et al., 2017).

We went on to test the involvement of the L-type Ca^2+^ channel itself in memory by knocking down its expression with *RNAi*. Knock-down of *Ca-*α*1D* in the M4/6 neurons abolished one-hour memory (p_Gal__4_ = 0.03, p_UAS_ = 0.007, Figure 1B). These animals exhibited no sensorimotor defects (Supplementary Table S1), verifying the observed phenotype as a genuine memory defect. In alignment with the lack of effect of nimodipine on the Ca^2+^ transient in control neurons, knock-down of the L-type channel had no effect on KCl-evoked Ca^2+^ influx in M4/6 cells (*p* = 0.6, Figure 2B).

We sought to resolve whether there was a behavioral interaction between 0N4R tau and the L-type Ca^2+^ channel by testing whether knocking down *Ca-*α*1D* could ameliorate the effect of tau on memory as it did on Ca^2+^ signaling. Strikingly, even though expression of human tau or *Ca-*α*1D-RNAi* removed memory alone, co-expression in M4/6 neurons yielded flies with memory performance indistinguishable from wild-type animals (p_Gal__4_ > 0.9, p_UAS_ > 0.9, Figure 1B). We tested whether this restoration of memory could have been a consequence of *Gal4* dilution due to the presence of multiple transgenes. Expression of an innocuous gene, *UAS-GFP*, with either *UAS-tau* or *UAS-Ca-*α*1D-RNAi* yielded animals with impaired memory (*p* = 0.02 and 0.005, respectively, Supplementary Figure S1). This demonstrates that dilution of transgene expression is not responsible for the observed change in memory performance. Lastly, the expression of transgenes in the M4/6 output neurons had no effect on the animals’ naïve sensorimotor behavior indicating that the effects seen in memory are not due to defective sensory responses (Supplementary Table S1).

To test if the apparent interaction between tau and L-type Ca^2+^ channels occurred in other neurons, we performed Ca^2+^ imaging of the entire MB (*OK107-Gal4*). The KCl-evoked Ca^2+^ response in this large population of neurons was reduced by the expression of tau (48.1% reduction, *p* = 0.006, Figure 2D; Higham et al., 2019), likely reflecting a reduction in excitability. MB neuronal architecture appeared grossly intact in tau-expressing animals (data not shown), and neurodegeneration has been shown not to play a significant role in memory defects at this age (Higham et al., 2019). In alignment with observations in M4/6 neurons, the expression of *Ca-*α*1D-RNAi* in the MB abolished one-hour memory (p_Gal__4_ = 0.001, p_UAS_ = 0.04, Figure 1A) but did not affect the magnitude of the Ca^2+^ transient in these neurons (*p* = 0.4, Figure 2D). The co-expression of these two transgenes in the MB resulted in a Ca^2+^ transient which was no different in magnitude compared to control animals (*p* > 0.9, Figure 2D). The ablation of one-hour memory caused by tau or *Ca-*α*1D-RNAi* expression in MB neurons was also reversed by co-expression of both transgenes as these animals exhibited a memory performance equivalent to control animals (p_Gal__4_ > 0.9, p_UAS_ > 0.9, Figure 1A).

**FIGURE 3:**
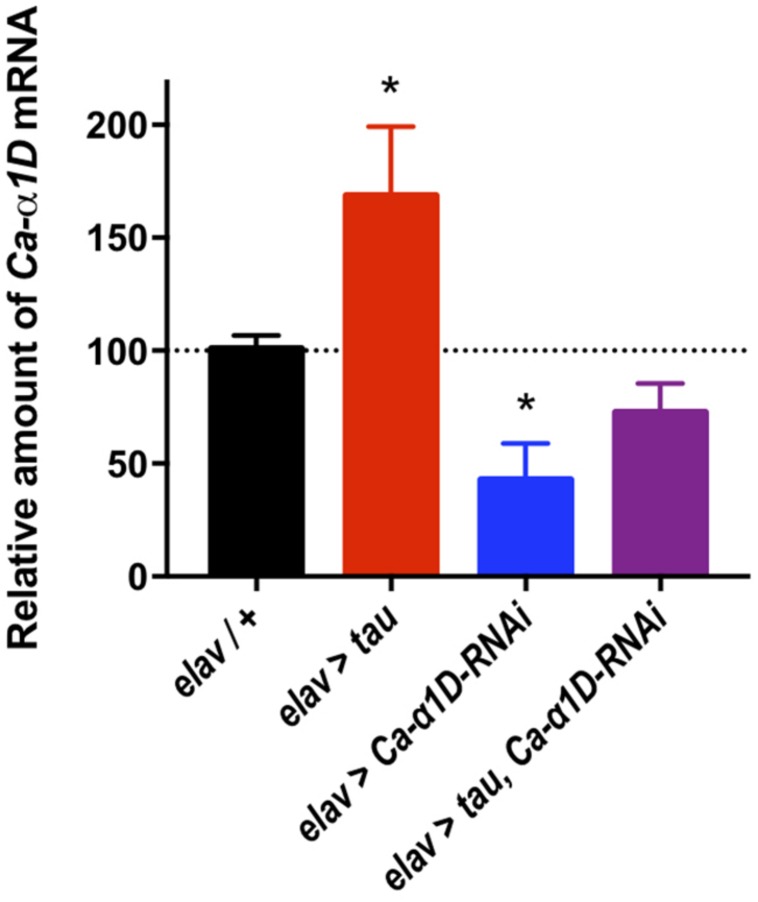
Tau expression increased *Ca^2+^ channel*α*1D mRNA* levels. Pan-neuronal Tau expression led to a 68% increase in *Ca-*α*1D mRNA* levels compared to controls, while expressing a *RNAi* against *Ca-*α*1D* reduced its expression by 58%. Co-expression of the *RNAi* in tau expressing flies partially restored levels of *Ca-*α*1D* compared to controls. *n* = 6 biological replicates of 23 or more heads each for all groups. Data analyzed with one-way ANOVA with Holm-Sidak’s *post hoc* tests. ^∗^*p* < 0.05.

The mechanism underlying tau-mediated augmentation of Ca^2+^ influx through L-type channels is not clear. It could be due to elevated expression of the *Ca-*α*1D* gene, increased trafficking to, or reduced recycling from, the plasma membrane or changes in single channel properties such as conductance or open probability. To investigate this further, we measured *Ca-*α*1D* expression in whole brain extracts by RT-qPCR. All transgenes were expressed in all neurons (*elav-Gal4*) to ensure changes in gene expression were detectable (Figure 3). *Ca-*α*1D-RNAi* knock-down reduced expression of the channel by 58% (*p* < 0.05), while expression of tau lead to a 68% (*p* = 0.04) increase in *Ca-*α*1D*. Interestingly, co-expression of tau and *Ca-*α*1D-RNAi* restored *Ca-*α*1D* to 72% (*p* = 0.3) of control fly levels. This strongly suggests that the effects observed in behavior and Ca^2+^ imaging are due to a change in *Ca-*α*1D* expression in the brain.

## Discussion

Selectively expressing tau in MB neuronal subsets revealed a non-specific adverse effect on memory processing (Figure 1). MB neurons sub-serving specific memory phases rely on different signaling molecules. For example, CaMKII is required in α′β′ neurons, but not αβγ, and vice versa for KCNQ channels (Cavaliere et al., 2013; Malik et al., 2013). This suggests that tau either promiscuously interacts with and disrupts numerous intracellular components or disrupts a pathway common to all MB neurons. *In vivo* Ca^2+^ imaging of different MB neuronal subsets has revealed the importance of Ca^2+^ transient plasticity in olfactory associative memory (Yu et al., 2006; Sejourne et al., 2011). Following conditioning, MB neurons exhibit differential Ca^2+^ responses to the CS+ and CS- odors (Yu et al., 2006; Sejourne et al., 2011). In the β′ lobe dendrites of M4/6 neurons, exposure to an aversively conditioned CS+ odour results in a greater Ca^2+^ influx compared with CS-, with the reverse being true for appetitively conditioned odors (Owald et al., 2015). Other MB neurons display a similar distinction between CS+ and CS-, with these differences believed to coordinate avoidance or approach behavior. Given that tau expression aberrantly elevated stimulus-evoked Ca^2+^ influx, it is plausible that this interferes with conditioning-induced Ca^2+^ transient plasticity. Augmented Ca^2+^ entry via L-type channels in tau-expressing neurons may ablate the difference in Ca^2+^ influx between CS+ and CS-, rendering them indistinguishable at the cellular level.

The knock-down of the *Drosophila* L-type Ca^2+^ channel demonstrated its importance in both the MB and the M4/6 neurons for memory. This aligns with data from Ca_v_1.2 knock-out mice, which are deficient in spatial memory tasks (Moosmang et al., 2005; White et al., 2008). Despite *R21D02, GCaMP6f* > *Ca-*α*1D-RNAi* and *OK107* > *Ca-*α*1D-RNAi* flies being memory deficient, the M4/6 and MB neurons of these animals displayed no reduction in evoked Ca^2+^ entry, nor did nimodipine have any effect on the Ca^2+^ transient in control M4/6 neurons (Figure 2). This suggests that perhaps only a small population of L-type channels is present in these neurons. A previous electrophysiological study of Ca^2+^ currents in cultured *Drosophila* giant embryonic neurons revealed only a very small block by nifedipine, likely reflecting a low number of L-type channels (Peng and Wu, 2007). Likewise an electrophysiological and pharmacological study of the adult *OK107-Gal4* MB neurons again showed only a small contribution of L-type channels to their Ca^2+^ transients (Jiang et al., 2005). Fluorescence imaging using GCaMP may not be sufficiently sensitive to resolve such a small contribution to global Ca^2+^ influx – a contribution that is nonetheless important for cellular function and, hence, memory.

It is not known whether the L-type channel plays any role in the plasticity of Ca^2+^ transients in *Drosophila per se*. However, these channels are vital for the function of memory-associated neurons. The generation of medium and slow afterhyperpolarizations (AHPs), which are a period of prolonged hyperpolarized membrane potential following action potential firing, in hippocampal neurons is dependent on Ca^2+^ entry through L-type channels (Lancaster and Adams, 1986; Marrion and Tavalin, 1998; Bowden et al., 2001). Elevated Ca^2+^ entry would augment AHPs and suppress neuronal firing, thereby disrupting neural circuit function and the behavior subserved by that circuit. This is apparent in mice and rabbits, which exhibit an age-related memory decline with concomitant elevation of L-type Ca^2+^ channel expression and AHP magnitude in the hippocampus (Moyer et al., 1992; Nunez-Santana et al., 2014). This impairs stimulus-evoked changes in neuronal activity which underpin learning (Moyer et al., 2000). Blocking augmented AHPs in aged rabbits with nimodipine facilitated enhanced performance in associative learning tasks (Straube et al., 1990). L-type Ca^2+^ channels also negatively regulate neuronal excitability in *Drosophila* neurons (Worrell and Levine, 2008), and so their augmentation by tau may exert a similar effect on *Drosophila* neurons to that observed in aging mammals, with their knock-down resulting in increased firing of neurons (Kadas et al., 2017).

In M4/6 neurons, we observed raised evoked Ca^2+^ influx due to tau expression, while Ca^2+^ influx was suppressed in MB neurons (Figures 2B,D). Augmentation of the Ca^2+^ transient in single tau-expressing M4/6 neurons reflects the elevated Ca^2+^ influx through L-type channels, as the transients were reduced by nimodipine and L-type channel knock-down. However, in a large population of MB neurons (∼2500 neurons) the elevated Ca^2+^ influx through L-type channels is likely small relative to the reduced Ca^2+^ levels due to suppressed neuronal activity, therefore a reduction in the Ca^2+^ transient was observed. The reduced excitability of MB neurons was rescued by co-expression of *Ca-*α*1D-RNAi* as this manipulation opposes the suppression of excitability caused by tau (Kadas et al., 2017). Not only were the tau-induced Ca^2+^ signaling defects rescued by manipulation of L-type Ca^2+^ channels, but so was olfactory memory (Figures 1A,B). This lends further credence to the suggestion that tau interacts with the L-type Ca^2+^ channel and also shows that this interaction solely mediates memory dysfunction, at least in young animals.

Raised L-type Ca^2+^ channel expression has also been documented in other AD models, as well as in aging. In agreement with our data, L-type Ca^2+^ current density was elevated in CA1 neurons of 3 × Tg mice (Wang and Mattson, 2014), as were AHPs in the dorsomedial entorhinal cortex of rTg4510 mice (Booth et al., 2016a). Our data shows that wild-type 0N4R tau, as well as the frontotemporal dementia-associated P301L mutant expressed in 3 × Tg and rTg4510 mice, is capable of augmenting Ca^2+^ influx in neurons. What is more, elevated expression of a mammalian L-type Ca^2+^ channel (Ca_V_1.2) was observed in a neuroblastoma cell line following transgenic Aβ_42_ expression; this resulted in reduced cell viability and six-fold increase in Ca^2+^ influx, that could be ameliorated by the L-type Ca^2+^ channel dihydropyridine blockers, nimodipine and isradipine (Copenhaver et al., 2011). This study went on to show that the L-type channel blocker, isradipine could increase survival of *Drosophila* overexpressing human amyloid precursor protein (APP_695_), as well as decreasing the accumulation of Aβ and phosphorylated tau in the triple transgenic AD mice (3 × TgAD) which express human Presenilin 1_M__146__V_, APP_Swedish_ and tau_P__30__L_. This indicates that correcting defective Ca^2+^ handling in AD may be of therapeutic benefit, particularly as L-type Ca^2+^ channels appear to be relevant in the human AD brain, too.

Raised L-type Ca^2+^ channel expression has been documented in the brains of AD patients (Coon et al., 1999), with increased L-type channels thought to underlie the memory loss and neurodegeneration that occurs in dementia (Missiaen et al., 2000; Mattson and Chan, 2001; Canzoniero and Snider, 2005; Thibault et al., 2007). Our data shows that there is increased expression of the *Drosophila* L-type Ca^2+^ channel, *Ca-*α*1D*, in tau expressing neurons, therefore suggesting conserved mechanisms of Aβ and tau-related calcium deficits across species (Figure 3). Importantly, clinical trials of L-type blockers show a slowing of cognitive decline in AD patients (Anekonda et al., 2011; Goodison et al., 2012; Nimmrich and Eckert, 2013).

In summary, using behavioral, physiological, pharmacological and molecular methods, we show that knock-down of the *Drosophila* L-type Ca^2+^ channel *Ca-*α*1D* can rescue tau mediated olfactory learning deficits by restoring Ca^2+^ handling in MB neurons.

## Materials and Methods

### *Drosophila* Genetics

Flies were raised at a standard density with a 12 h:12 h light dark (LD) cycle on standard *Drosophila* medium (0.7% agar, 1.0% soya flour, 8.0% polenta/maize, 1.8% yeast, 8.0% malt extract, 4.0% molasses, 0.8% propionic acid, and 2.3% nipagen) at 25°C. Wild-type control was *Canton S w-* (*CSw-*) and *R21D02-GAL4, UAS-GCaMP6f* were kind gifts from Prof. Scott Waddell (University of Oxford). *UAS-human MAPT (TAU 0N4R) wild-type* (Wittmann et al., 2001; Kerr et al., 2011) was a kind gift from Prof. Linda Partridge (University College London), *UAS-GFP* was a gift from Prof. Mark Wu (John Hopkins University). The following flies were obtained from the Bloomington and Vienna fly stock centers: *OK107-Gal4* (Bloomington *Drosophila* stock center number BDSC:854), *c305a-Gal4* (BDSC:30829), *amn(c316)-Gal4* (BDSC:30830), *MB247-Gal4* (BDSC:50742), *elav-Gal4* (BDSC:8760), *UAS-GCaMP6f* (BDSC:42747) and *UAS-Ca-*α*1D-RNAi* flies [Vienna *Drosophila* resource center GD51491 (Kadas et al., 2017)].

### Aversive Olfactory Conditioning

All memory experiments were carried out at 25°C and 70% relative humidity under dim red light. Flies were used for experiments after 2–5 days of aging at 25°C and 70% relative humidity in a 12-hour light: 12-hour dark environment. Using a previously published protocol (Mershin et al., 2004; Krashes et al., 2007; Kosmidis et al., 2010; Malik et al., 2013; Malik and Hodge, 2014; Barnstedt et al., 2016), groups of 25–50 flies were first transferred from food tubes into the training tube lined with an electrifiable grid. After acclimatization to the electrified tube for 90 s, flies were exposed to either 3-octanol (OCT, Sigma) or 4-methylcyclohexanol (MCH, Sigma) (conditioned stimulus, CS+) paired with twelve 70 V DC electric shocks (unconditioned stimulus, United States) over 60 s (shocks of duration 1.25 s with inter-shock latency of 3.75 s). This was followed by a 30 s rest period with no stimulus. Flies were then exposed to the reciprocal odour (CS−) for 60 s with no electric shock. Memory retention was tested one-hour post-conditioning (intermediate-term memory). To account for any innate bias the flies may have towards an odour, the CS + odour was reversed in alternate groups of flies and the performances from these two groups averaged to give *n* = 1. Moreover, the order of delivery of CS+ and CS− was alternately reversed.

To test memory, flies were placed at the choice point of a T-maze with one pathway exposed to CS+ and the other to CS−. After 120 s, the number of flies choosing each pathway was counted. Memory was quantified using the performance index (PI):

$$P\,I=\frac{N_{C\,S-}-N_{C\,S+}}{N_{C\,S-}+N_{C\,S+}}$$

where N_*CS–*_ and N_*CS+*_ is the number of flies choosing CS− and CS+, respectively. A *PI* = 1 indicates perfect learning where all flies chose CS−, and *PI* = 0 indicates a 50:50 split between CS− and CS+ and, therefore, no learning.

### Calcium Imaging

GCaMP imaging was performed using previously published protocols (Cavaliere et al., 2012; Gillespie and Hodge, 2013; Malik et al., 2013; Schlichting et al., 2016; Shaw et al., 2018) with flies being anaesthetized on CO_2_, decapitated and their brains dissected out of the head in extracellular saline containing (in mM): 101 NaCl, 1 CaCl_2_, 4 MgCl_2_, 3 KCl, 5 D-glucose, 1.25 NaH_2_PO_4_, and 20.7 NaHCO_3_ adjusted to a final pH of pH 7.2 with an osmolality of 247–253 mmol/kg. Brains were held ventral side up in a recording chamber using a custom-made anchor and visualized with a 40× water-immersion lens on an upright microscope (Zeiss Examiner Z1).

Brains were superfused with extracellular saline (5 mL/min) as above and cells were depolarized by bath application of 100 mM KCl in extracellular solution (362 mmol/kg) for 15 s at 5 mL/min. Drug-containing or Ca^2+^-lacking solutions were superfused over the brain for 60 s prior to imaging. The Ca^2+^-lacking external solution contained 8 mM MgCl_2_.

Images were acquired at 8 Hz with 50 ms exposure using a CCD camera (ZEISS Axiocam) and a 470 nm LED light source (Colibri, ZEISS) and recorded with ZEN (Zeiss, 4 frames/sec). Baseline fluorescence (F_0_) was taken as the mean fluorescence during the 10 s (80 images) prior to the start of KCl perfusion. The change in fluorescence relative to baseline [(F-F_0_)/F_0_, where F is fluorescence at a given time] following KCl addition was recorded, and the peak change [(F_*max*_-F_0_)/F_0_] was used as a metric of transient [Ca^2+^] increase. Example traces were plotted using Origin 9 (Origin Lab).

All chemicals were purchased from Sigma-Aldrich (Gillingham, United Kingdom), except for nimodipine which was purchased from Tocris (Bristol, United Kingdom) and amiloride which was donated by Prof. David Sheppard (University of Bristol).

### RT-qPCR

Relative measure of Ca-α1D expression levels was assessed by two-step qPCR. 2–5 days old male flies were anesthetized with CO_2_ and decapitated, obtaining six biological replicates with 23 heads each. Total *RNA* was extracted from head lysates by organic phenol/chloroform method using TRIzol reagent (Invitrogen). *RNA* quantification was carried out in Nanodrop spectrophotometer (Thermo Scientific) and *RNA* integrity was checked by electrophoresis in 1% agarose gel. Samples were treated with TURBO DNA-free kit (Invitrogen) in order to remove genomic DNA contamination. Reverse transcription was carried out using RevertAid First Strand cDNA Synthesis Kit (Thermo Scientific) following manufacturer’s instructions, with 500 ng of *RNA* as template and Oligo (dT) as primer to amplify total *mRNA*. cDNA samples were stored at −20°C or used immediately for qPCR reactions.

Quantitative PCR reactions were carried out in QuantStudio 3 Real-Time PCR system (Applied Biosystems) using HOT FIREPol EvaGreen qPCR Mix Plus (Solis BioDyne). The primers used to amplify Ca-α1D *mRNA* were as follows: Ca1DF 5′-CCTTGAGGGCTGGACTGATG-3′ and Ca1DR 5′-ATCACGAAGAAGGCACCCAG-3′ with a PCR product expected size of 108 bp and 104% primer efficiency (Supplementary Figure S3). As a housekeeping gene, the following primers for GAPDH2 *mRNA* were used: GAPDH2F 5′-CGTTCATGCCACCACCGCTA-3′ and GAPDH2R 5′-CCACGTCCATCACGCCACAA-3′. The expected PCR product size was 72 bp and the primer efficiency was 100%. To activate DNA polymerase, a first step of 15 min at 95°C was used, followed by 50 cycles of 30 s at 95°C, 30 s at 60°C, followed by a 1 min 72°C elongation step. At the end of the experiment, a temperature ramp from 60°C to 95°C was used for melting curve analysis and the product fit to the predicted melting curve obtained by uMelt software (Dwight et al., 2011). Quantification for each genotype and each gene was carried out using the 2^(–Δ^ ^Δ^ ^Ct)^ method and data was expressed as a percentage of change.

### Analysis

All data were analyzed using Prism 7 (GraphPad Inc.). Data were scrutinized to check they met the assumptions of parametric statistical tests, and non-parametric, rank-based alternatives were used where appropriate. Details of statistical tests used are in figure legends. Data are presented as mean ± standard error of mean.

## Data Availability

The raw data supporting the conclusion of this manuscript will be made available by the authors, without undue reservation, to any qualified researcher.

## Author Contributions

All authors devised and performed the experiments, and wrote and edited the manuscript. JH secured the funding.

## Conflict of Interest Statement

The authors declare that the research was conducted in the absence of any commercial or financial relationships that could be construed as a potential conflict of interest.
