# Supplementary material for: Restoration of Olfactory Memory in Drosophila Overexpressing Human Alzheimer’s Disease Associated Tau by Manipulation of L-Type Ca2+ Channels
Source: Front Cell Neurosci. 2019 Sep 10;13:409. doi: 10.3389/fncel.2019.00409 (PMC6746915; doi:10.3389/fncel.2019.00409)
Supplement: Supplementary file 1 [file Presentation_1.pdf]

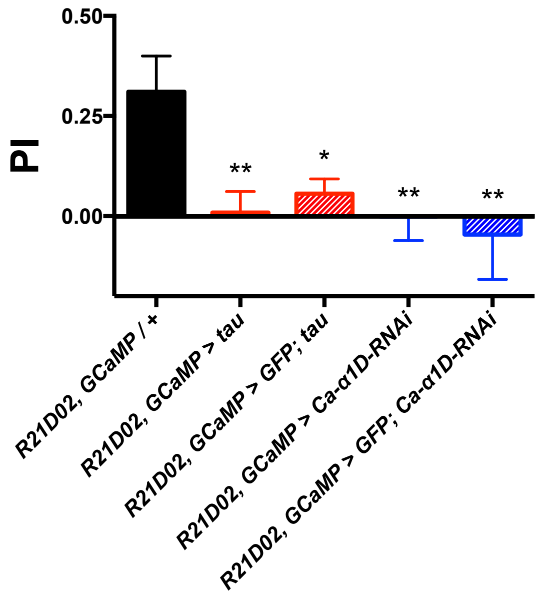

Figure S1: Control memory experiments. Gal4 dilution was not responsible for restoration of memory in double transgene flies. Co-expression of either tau or *Ca-α1D-RNAi* with the innocuous transgene GFP resulted in animals with impaired memory ($p<0.0001$) not significantly different to tau or *Ca-α1D-RNAi* expressed on their own ($p=0.965$ and $p=0.74$, one-way ANOVA). Note that *R21D02, GCaMP / +*, *R21D02, GCaMP* > *tau* and *R21D02, GCaMP* > *Ca-α1D-RNAi* are the same as in Fig 1B.

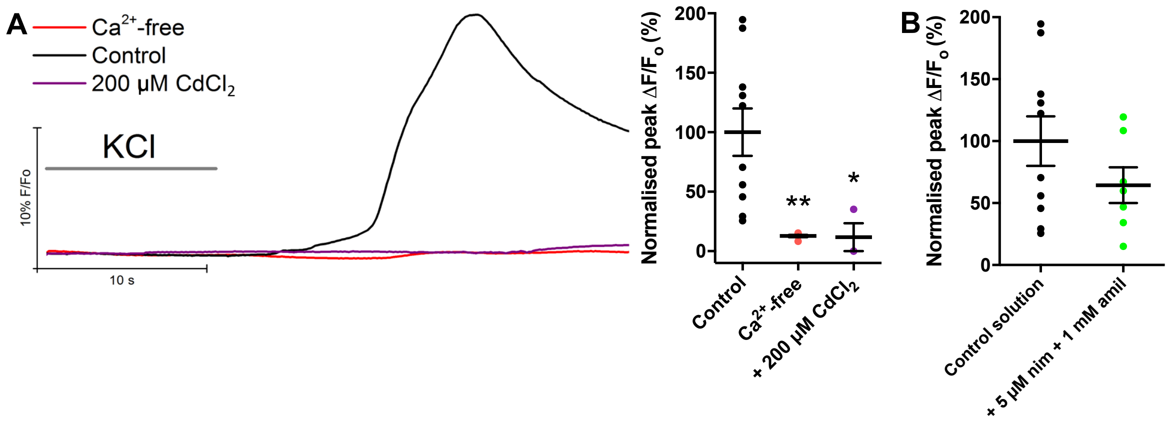

Figure S2: Ca^{2+} transients in control M4/6 neurons rely on influx through nimodipine- and amiloride-insensitive Ca^{2+} channels. (**A**, left) Example fluorescence trace showing that omission of Ca^{2+} from the bath solution (red) or addition of 200 µM cadmium (purple) ablated the Ca^{2+} transient. (**A**, right) Grouped data from these experiments (Kruskal-Wallis test). (**B**) Addition of 5 µM nimodipine and 1 mM amiloride to the bath solution did not reduce the peak magnitude of the Ca^{2+} transients (t-test).

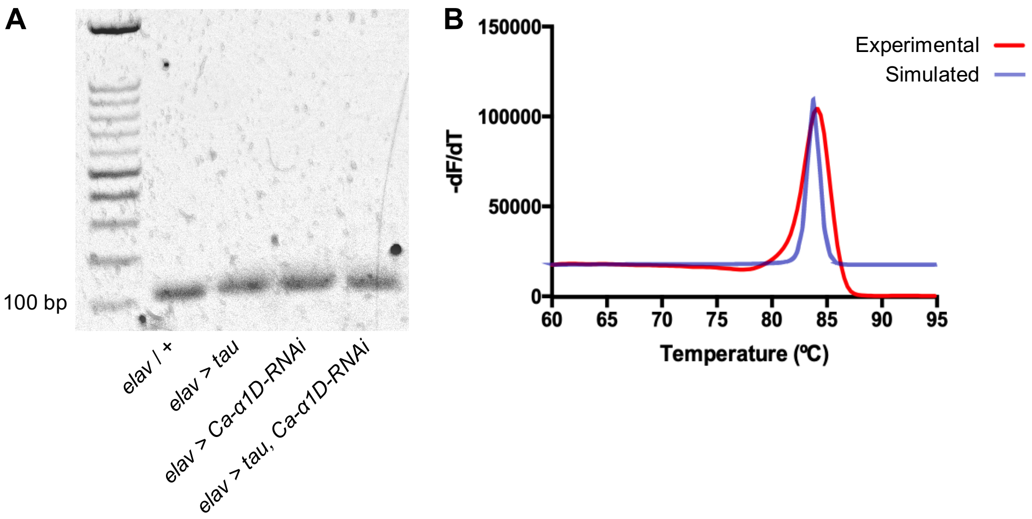

Figure S3. Primer specificity for *Ca-α1D* quantitative PCR. (**A**) The obtained qPCR reaction product of the expected size of 108 bp is observed in all genotypes. (**B**) The observed temperature dependent fluorescence change was used for melting curve analysis. A single product was obtained (red line) and fit with the predicted melting curve obtained by uMelt software (blue (Dwight et al., 2011)).

Table S1: Tau or *Ca-α1D-RNAi* expression did not cause sensorimotor defects. Tau or *Ca-α1D-RNAi* did not affect avoidance of shock, 3-octanol (Oct) or 4-methylcyclohexanol (MCH).

genotype	% Shock avoidance (mean ± SEM)	Oct avoidance (mean ± SEM)	MCH avoidance (mean ± SEM)
OK107 / +	83.6 ± 6.2	0.76 ± 0.05	0.77 ± 0.08
OK107 > tau	85.3 ± 2.6	0.82 ± 0.04	0.70 ± 0.01
OK107 > Ca-α1D-RNAi	81.0 ± 2.3	0.64 ± 0.04	0.73 ± 0.02
OK107 > tau, Ca-α1D-RNAi	94.0 ± 2.3	0.74 ± 0.13	0.83 ± 0.10
R21D02, GCaMP / +	79.1 ± 2.1	0.60 ± 0.12	0.43 ± 0.16
R21D02, GCaMP > tau	79.2 ± 4.6	0.64 ± 0.08	0.55 ± 0.02
R21D02, GCaMP > Ca-α1D-RNAi	83.0 ± 1.2	0.68 ± 0.04	0.74 ± 0.06
R21D02, GCaMP > tau, Ca-α1D-RNAi	81.2 ± 2.6	0.45 ± 0.09	0.44 ± 0.07
R21D02, GCaMP > GFP; tau	84.3 ± 4.9	0.77 ± 0.13	0.56 ± 0.07
R21D02, GCaMP > GFP; Ca-α1D-RNAi	93.7 ± 1.9	0.69 ± 0.06	0.85 ± 0.02
c305a / +	80.9 ± 3.4	0.60 ± 0.12	0.75 ± 0.04
c305a > tau	80.0 ± 4.4	0.77 ± 0.05	0.77 ± 0.04
MB247 / +	77.3 ± 2.5	0.71 ± 0.05	0.66 ± 0.06
MB247 > tau	71.3 ± 6.5	0.85 ± 0.07	0.88 ± 0.05
amn(c316) / +	83.2 ± 2.9	0.60 ± 0.04	0.65 ± 0.02
amn(c316) > tau	81.1 ± 0.8	0.77 ± 0.04	0.75 ± 0.05
tau / +	97.0 ± 1.3	0.79 ± 0.06	0.89 ± 0.04
Ca-α1D-RNAi / +	94.0 ± 2.1	0.58 ± 0.05	0.67 ± 0.17
tau, Ca-α1D-RNAi / +	97.8 ± 1.3	0.69 ± 0.12	0.59 ± 0.09
